# Changes of physicochemical and functional properties of processed cheese made with natural cheddar and mozzarella cheeses during refrigerated storage

**DOI:** 10.1038/s41598-024-53748-z

**Published:** 2024-02-14

**Authors:** Tongji Liu, Jingwei Wu, Tariq Aziz, Rui Xue, Manal M. Khowdiary, Zhennai Yang

**Affiliations:** 1 Key Laboratory of Geriatric Nutrition and Health of Ministry of Education, Beijing Technology and Business University, Beijing, 100048 China; 2 Beijing Engineering and Technology Research Center of Food Additives, Beijing Technology and Business University, Beijing, 100048 China; 3 Department of Chemistry, Al-Leith University College, Umm Al Qura University, Makkah, Saudi Arabia

**Keywords:** Processed cheese, Cheddar, Mozzarella, Stretchability, Fat leakage, Microbiology, Bacteria, Industrial microbiology

## Abstract

The present study aimed to investigate changes of physicochemical and functional properties of the processed cheeses (PCs) made with Cheddar (PC1), Mozzarella (PC2) and both of them at a ratio of 1:1 (PC3) during storage at 4 °C for 4 months. The results showed that the type of natural cheese used affected the composition of PCs with lower fat content in PC2 due to the lower fat content of Mozzarella cheese used. PC2 with lower fat content showed decreased meltability and oil leakage compared with PC1 and PC3. The stretchability of all the samples significantly (*P* < 0.05) decreased during storage, and PC1 showed lower stretchability. This was confirmed by increased protein hydrolysis of all the samples during the storage with a higher level of proteolysis in PC1, leading to decreased stretchability of PCs. Further low-field nuclear magnetic resonance analysis indicated more entrapped water in cheese due to moisture migration into the cheese matrix that might squeeze the fat globules to aggregate, causing more fat leakage during later stages of storage. This was evidenced by microstructural analysis showing different extents of increase in fat particle sizes and decrease in free serum in all the PC samples over the storage time. Therefore, the present study provides further understanding of the mechanism of quality change of PC during refrigerated storage as affected by proteolytic properties and composition of natural cheese used.

## Introduction

Cheese is a highly demanded dairy product that can be consumed directly or used as an ingredient in sandwiches, pizzas, corn kernels and other products^1^. Natural cheese, nevertheless, presents sophisticated making processes and low stability, posing significant storage and transportation challenges, especially in areas far from milk sources^2^. Processed cheese (PC) made with natural cheese, casein, butter as well as other dairy and nondairy ingredients under thermal heating conditions is generally more stable than natural cheese, and PC has become a common alternative to natural cheese^3^. Although the quality of PC can be maintained by regulating raw materials and processing parameters independent of seasons and origins, prolonged refrigeration storage of PC often leads to some undesirable changes such as poor meltability, excessive fat leakage and texture defects^4^. Consequently, increasing concerns for PC have been observed regarding the physicochemical changes of PC during refrigerated storage to improve the quality of their end-use applications.

Meltability, free-oil formation and stretchability are considered as critical heat-induced functionalities of cheese^5^. During heat processing of PC, fat is considered the only substance in cheese that can be completely melted, providing the driving force of cheese melting^6^. Maintaining stability of fat within the protein matrix structure in PC during storage is important to prevent formation of large spherical fat droplets and accumulation as oil pockets or pools, notably on the surface^7^. As the major component of PC, the type of natural cheese used had a significant effect on the quality of PC^8^. Since Cheddar and Mozzarella are the two most consumed cheeses worldwide, they are frequently used as the natural cheeses for making PC with generally more acceptable sensory properties^9,10^. As a type of ripened cheese, Cheddar cheese contains various proteolytic activities that may endure the heating process of PC and cause protein degradation in PC during storage^11^. For the unripened Mozzarella cheese, the thermal–mechanical stretching step during the processing largely inactivated the proteolytic enzymes in cheese^12^. Combination of these 2 types of cheese in making PC may provide different taste, flavor, and texture, satisfying different consumer needs to support pizza marketing^11^. Although various proteins^13^, fats^14^, hydrocolloids^15^, and processing parameters^16^ have been studied for their effects on the heat-induced functionalities of cheeses, few studies are focused on the effect of natural cheeses such as Cheddar and Mozzarella cheeses on the physicochemical changes of PC during storage.

The objective of this study was to investigate changes of the physiochemical and functional properties of PC prepared from Cheddar and Mozzarella cheeses, as well as their mixture, focusing on the changes of meltability, stability of fat, stretchability, proteolysis, moisture distribution and microstructure of PC during refrigerated storage. The present study provides further understanding on the mechanism of physicochemical changes affecting the refrigerated storage stability of PC with technological significance for improvement of the quality of PC.

## Materials and methods

### Materials

In addition to distilled water, the following ingredients were used to prepare the processed cheese samples: (1) Cheddar cheese (26.3 wt% protein, 42.8 wt% moisture, 30.0 wt% fat) and Mozzarella cheese (25.3 wt% protein, 47.2 wt% moisture, 24.2 wt% fat) (Dairy Products Co. Ltd., Auckland, New Zealand); (2) rennet casein powder (Dairy Products Co. Ltd., Auckland, New Zealand); (3) palm oil and (4) whole milk powder (Bright Dairy &Food Co. Ltd., Shanghai, China); (5) sodium citrate, sodium hexametaphosphate and disodium hydrogen phosphate (Institute of Chemical Reagent, Beijing, China).

### Preparation of processed cheese

The formulations of different PC samples are shown in Table 1. A ZJR-5 Vacuum Homogenizer with direct steam injection was used to produce PC, according to the method by Li et al.^17^ with the following changes.

**Table 1:** Formulations of the experimental processed cheese (PC) samples.

Ingredient and parameter	Composition of PC (%, w/w)		
	PC1	PC2	PC3
Cheddar cheese	51	0	25.5
Mozzarella cheese	0	51	25.5
Rennet casein	8	8	8
Palm oil	13	13	13
Whole milk powder	3	3	3
Emulsifying salts	1	1	1
Potassium sorbate	0.1	0.1	0.1
Moisture	48–49	48–49	48–49
pH	5.8–5.9	5.8–5.9	5.8–5.9

Before production of PC, natural cheeses were tempered overnight at 4 °C and shredded using a knife. All materials except emulsified salts were transferred to the homogenizer and mixed at 90 rpm for 60 s. The blend stirred constantly at around 100 rpm and heated to 65 °C. Emulsified salts were dissolved in the remaining water and then transferred to the homogenizer. Subsequently, the vacuum was set to -6 Bar and the temperature increased from 65 to 85 °C, holding at 85 °C for 4 min. The pH of the final product was adjusted to pH 5.8 to 5.9 by adding lactic acid. After that the hot samples were poured into silastic molds cooled to 40 °C, then removed from the molds, and vacuum packaged. All the samples were stored at 4 °C for 4 months in a refrigerator (BCD-325WFPM, Midea, Midea Group Co., Ltd, Beijing, China).

### Compositional analysis

In the first day of the storage period, samples of PC were analyzed for moisture, fat, protein content and pH value according to Wang et al.^18^.

### Determination of meltability and stretchability

The meltability and stretchability of the PC samples collected at months 0, 1, 2 and 4 of the storage period were determined according to a described method^17^ with some modifications. The cheese samples were cut into a cylinder of 10 mm height and 20 mm diameter using a special punch and placed in a petri dish with filter paper. The samples were then heated in an oven (MG38CB-AA, Midea, Midea Group Co., Ltd, Guangdong, China) at 130 °C for 10 min. After cooling to room temperature, the diameters of the melted cheese were measured four times and the mean values were calculated. The cheese was covered with bread and heated at a temperature of 220 °C for 6 min, the bread was lifted after the cheese had cooled, and the instantaneous height of the sliced bread could be used as an indicator of the stretchability of the cheese. Each measurement was repeated three times.

### Free oil content

The free oil content of the PC samples collected at months 0, 1, 2 and 4 of the storage period was determined according to a described method^19^ with some modifications. Grated cheese (3 g) was weighed into a Gerber butyrometer and then immersed in boiling water for 15 min to melt the cheese. Methanol diluted in distilled water (1:1, 15 mL at about 55 °C) was immediately added in butyrometer and then the bottle was centrifuged using a Gerber centrifuge (NOVA-SAFETY, Funke Gerber Co. Ltd, Germany) at about 57.5 °C for 15 min. The free oil content of the cheese was expressed as a percentage as follows:

$${\text{Free oil }}\left( \% \right) = {\text{reading of fat/cheese weight}}.$$

### Determination of fat particle size

A sample of each PC (0.5 g) collected at months 0, 1, 2 and 4 of the storage period was placed in 50 mL of a solution (pH 10) containing EDTA (0.375%, w/w) and Tween 20 (0.125%, v/v) and refrigerated at 4 ℃ overnight before testing^20^^.^ The above solutions were equilibrated at room temperature for 1 h and then the particle size of the fat droplets in the cheese was determined using a SALD-2300 laser diffraction particle size analyzer (Shimadzu, Kyoto, Japan), and the results of the fat particle size measurements were expressed as D[4,3].

### Determination of plasmin (PL) and plasminogen (PLG) activity

PC samples collected at months 0, 1, 2 and 4 of the storage period were diluted 1:1 (v/v) with an assay buffer containing 0.1 M Tris–HCl, 8 mM EACA, 0.4 M NaCl, pH 8^21^. The cheese extracts and horseradish peroxidase (HRP) were added to the wells of micro-titer plate pre-coated with a tibodies for the enzymes (PL and PLG) and incubated at room temperature in dark for 60 min. After the washing step, addition of a colorless enzyme substrate tetramethylbenzidine (TMB) produces a colored reaction product. TMB turns blue in a reaction catalyzed by the HRP enzyme. This reaction was terminated by addition of sulphuric acid. The color change was measured spectrophotometrically at a wavelength of 450 nm using Infinite 200 Pro NanoQuant (Tecan, Switzerland).

### Determination pH 4.6-soluble nitrogen and intact casein content

The pH 4.6-soluble nitrogen and intact casein content of the PC samples collected at months 0, 1, 2 and 4 of the storage periods were determined. Cheese (0.75 g) was grated into a suspension, and 25 ml of pH 4.6 acetate buffer was added, and then held in a water bath at 40 °C for 1 h. The sample suspension was centrifuged at 10,000 g and 4 °C for 10 min. The supernatant was separated and analyzed by an automatic Kjeldahl nitrogen analyzer (Kjeltec 8400, FOSS Co. Ltd, Denmark). Soluble nitrogen was expressed as a percentage of total nitrogen.

Intact casein content of the cheese was expressed as a percentage as follows^22^:

$$\mathrm{Intact casein content }\left({\%}\right)=\frac{\left(\mathrm{Total nitrogen}-\mathrm{pH }4.6\mathrm{ soluble nitrogen}\right)}{\mathrm{Total nitrogen}}\times 100$$

### Low-field nuclear magnetic resonance (LF-NMR)

LF-NMR relaxation tests (MicroMR12-025V Niumag Corporation, Suzhou, China) were performed according to a previously described method^23^ with some modifications. The PC samples collected in the 1st, 2nd and 4th months of the storage period were subjected to LF-NMR. Approximately 4 g of the cheese sample was transferred into an NMR tube with a diameter of 25 mm and inserted into a nuclear magnetic tube to measure the transverse spin–spin relaxation (T2) using the Carr-Purcell-Meiboom-Gill (CPMG) sequence by setting parameters that were: hard pulse 90-degree pulse width (P1) of 6 μs, hard pulse 180-degree pulse width (P2) of 10.24 μs, repeat sample waiting time (TW) of 4500 ms, echo time (TE) of 0.2 ms, number of echoes (NECH) of 5000, number of repeat samples (NS) of 4, receiver bandwidth (SW) of 250 kHz, RF delay time (RFD) of 0.8 ms analog gain (RG1) of 10 dB digital gain (DRG1) of 3, test temperature of 32 °C, and pulse signal acquisition. Finally, the resulting test data were inverted 10,000 times and plotted in the software to obtain the transverse relaxation time T2 profile taken in triplicate.

### Confocal laser scanning microscopy (CLSM)

The PC samples collected in the 1st, 2nd and 4th months of the storage period were subjected to CLSM. The grated cheese was thinly sliced using a scalpel blade. Samples were stained by placing 10 ml of mixed dye (1% fast green stain and 0.5% Nile red stain mixed in a 1:1 ratio) on a slide and immersing a thin section of cheese in the dye^24^. The dye was eluted and the slide was then placed on a TCS-SP8X confocal laser scanning microscopy (Leica, Germany) for examination. The excitation wavelengths of the aqueous solutions of Nile red and solid green were 528 nm and 633 nm, respectively.

### Statistical analysis

All measurements were conducted in 3 independent replicates. Statistical analysis was performed for variance (ANOVA) and significance (*P* < 0.05) using SPSS software 22.0.

## Results and discussion

### Cheese composition

Table 2 shows the composition of the PC samples prepared with Cheddar (PC1), Mozzarella (PC2) and both of the cheeses at a ratio of 1:1 (PC3). No significant (*P* > 0.05) differences were found in the moisture and protein contents, and the pH values between the cheeses. Compared with PC1 and PC3, PC2 had significantly (*P* < 0.05) lower fat content due to the lower fat content of Mozzarella cheese used. Gulzar et al.^25^ also found that the pizza cheese blend made with Mozzarella cheese had lower fat content than that made with Cheddar cheese.

**Table 2:** Main composition and pH of the processed cheeses made with Cheddar (PC1), Mozzarella (PC2) and both of them (1:1) (PC3).

Component and pH	PC1	PC2	PC3
Moisture content (g/100 g)	48.2^a^ ± 0.33	49.1^a^ ± 0.34	48.5^a^ ± 0.77
Protein content (g/100 g)	22.8^a^ ± 0.62	22.1^a^ ± 0.43	22.1^a^ ± 0.49
Fat content (g/100 g)	27.1^a^ ± 0.16	23.8^c^ ± 0.17	25.4^b^ ± 0.31
pH	5.91^a^ ± 0.02	5.96^a^ ± 0.04	5.93^a^ ± 0.04

^a-^^c^Means for the same cheese sample stored for different months without common letters are significantly different (*P* < 0.05).

### Meltability, fat leakage and stretchability of PC

**Figure 1:**
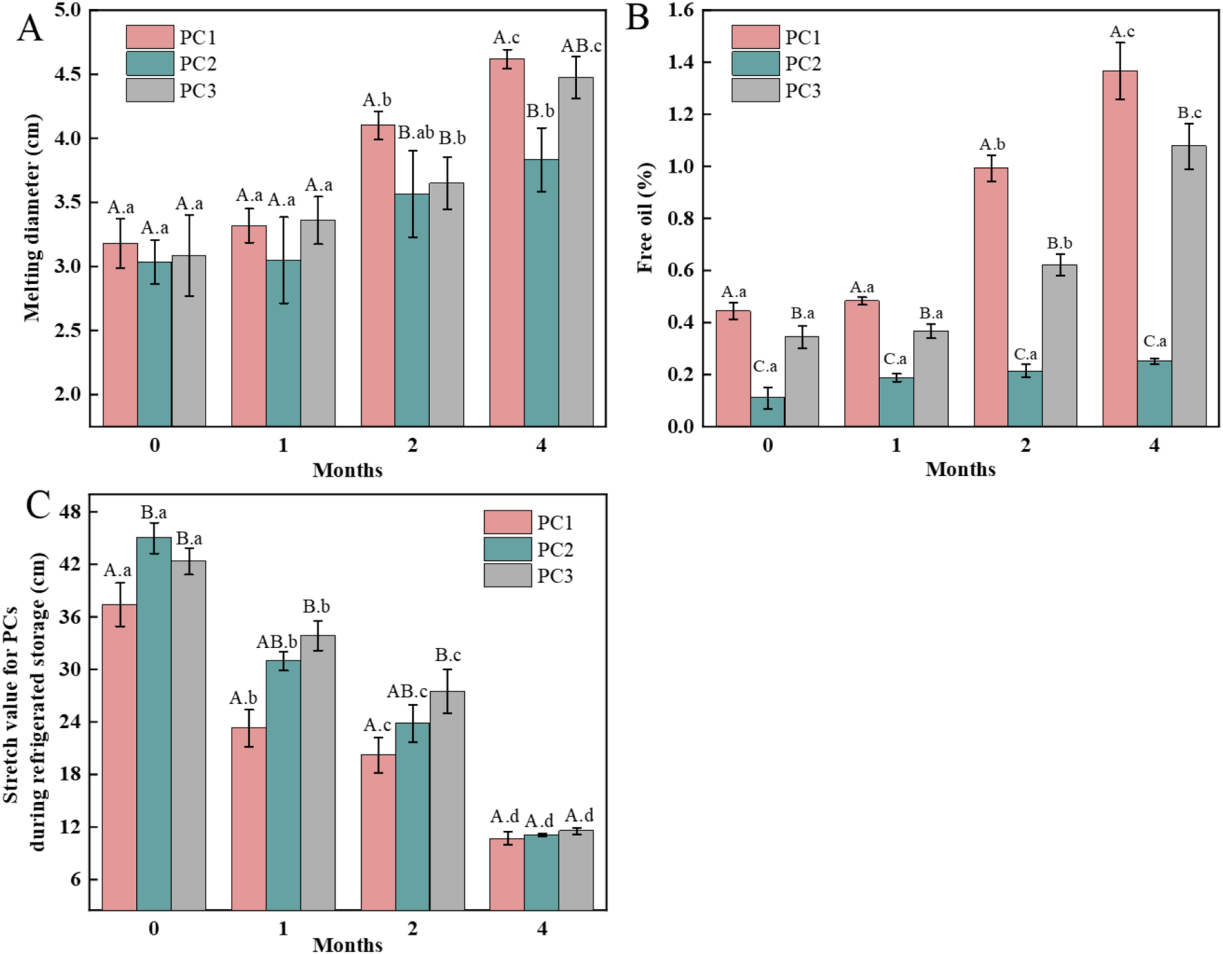
Changes of meltability (**A**), free oil content (**B**) and stretchability (**C**) of the processed cheeses made with Cheddar (PC1), Mozzarella (PC2) and both of them (1:1) (PC3) during 4 months of refrigerated storage. ^A-C^ means for cheese sample at the same storage time without common letters are significantly different (*P* < 0.05).^a-d^ means for the same cheese sample stored for different months without common letters are significantly different (*P* < 0.05).

Meltability, fat leakage and stretchability are attractive baking properties of PC, which enable PC to be used ideally for pizza topping, toasted sandwiches and other fast foods^26^. Decent free oil covering the surface of cheese was considered not only preventing the surface from dehydration, but also stimulating the consumer's appetite^27^. Change of meltability of the PC samples made with Cheddar (PC1), Mozzarella (PC2), and both of them (PC3) during 4 months of refrigerated storage is presented in Fig. 1A. In the beginning of storage, the meltability of all the cheese samples were not significantly (*P* > 0.05) different, but after 4 months of storage, PC1 (4.62 cm) and PC3 (4.48 cm) had significantly (*P* < 0.05) higher meltability than PC2 (3.83 cm). Concerning oiling-off of the samples (Fig. 1B), significant (*P* < 0.05) difference in the free oil content as determined for the 3 groups of PC during the storage was observed. PC1 had the highest fat leakage, while PC2 the lowest during all stages of the storage. Concerning stretchability of the samples (Fig. 1C), all the samples showed a significant (*P* < 0.05) decrease in stretch length over the storage time, and no significant (*P* > 0.05) difference was observed between the 3 groups of the samples after 4 months of storage, though PC2 and PC3 had higher stretch values than PC1 in the first 2 months of storage.

It was reported that meltability and fat leakage of cheese were closely related to its content of fat that acted as a lubricant promoting heat-induced cheese melting process, but higher fat content facilitated formation of larger anisotropic fat particles that further contributed to the melting and fat leakage of cheese^28,29^. The higher meltability and free fat content of PC1 of this study could be mainly due to its higher fat content (27.1%, w/w) than those of PC2 (23.8, w/w) and PC3 (25.4%, w/w) (Table 2). Moreover, the increased meltability and decreased stretchability of PC1 during storage might be associated with more degradation of casein in PC1, which reduced the total number of protein–protein bonds and the tensile strength of cheese upon protein hydrolysis^30,31^. The decreased protein–protein interactions was also reported to increase fat globules coalescence, resulting in oiling-off in pizza cheese made with semi-ripened Cheddar cheese with high proteolytic activity^22^. The presence of higher proteolytic activities in PC1 made with Cheddar cheese than those in PC2 and PC3 was discussed in the following Sects. (3.4).

### Plasmin activity and proteolysis of PC

**Figure 2:**
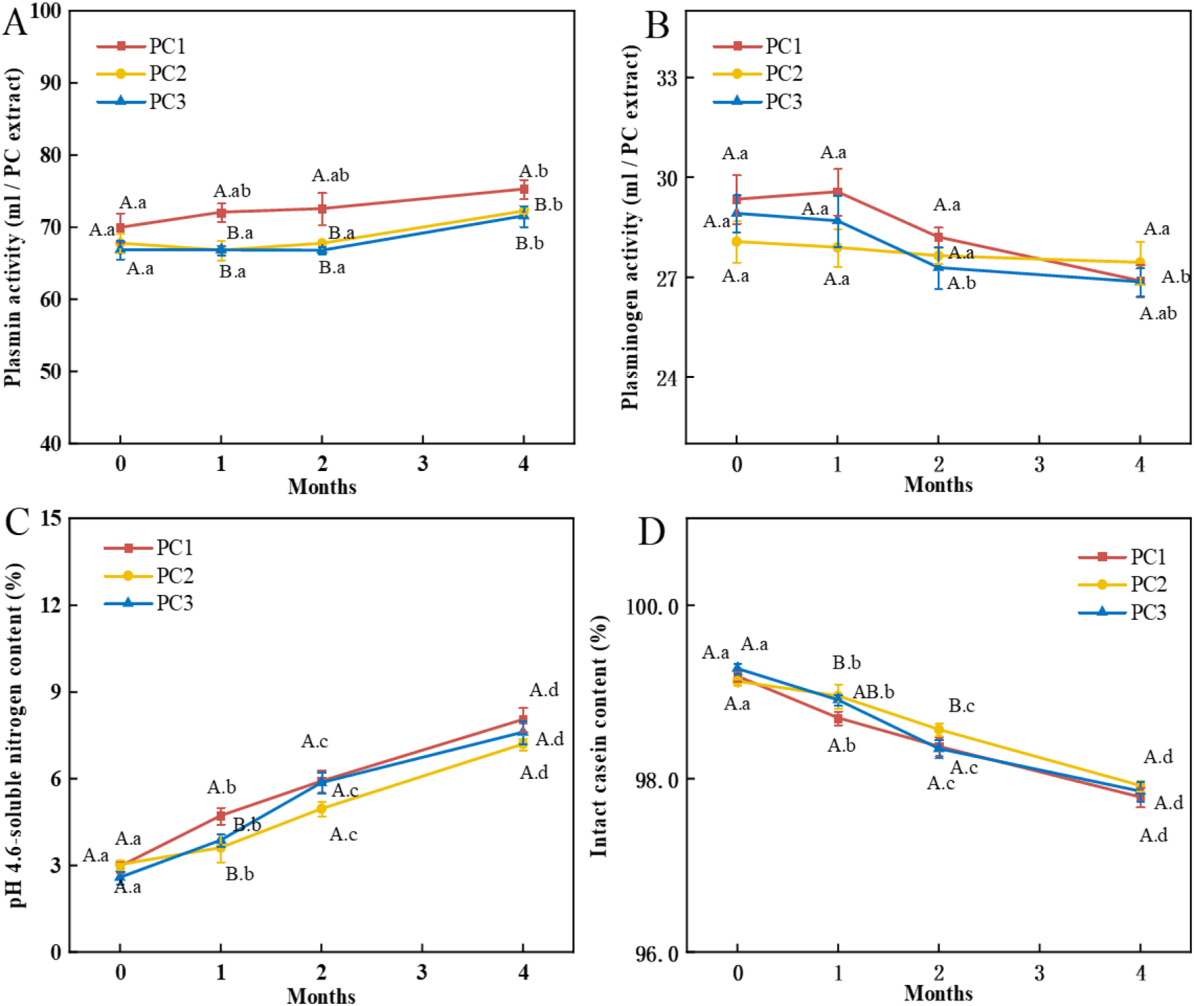
Changes of plasmin activity (mU) (**A**); plasminogen activity (mU) (**B**); pH 4.6-soluble nitrogen content (**C**) and intact casein content (**D**) in the processed cheeses made with Cheddar (PC1), Mozzarella (PC2) and both of them (1:1) (PC3) during 4 months of refrigerated storage.^A–B^ means for cheese samples at the same storage time without common letters are significantly different (*P* < 0.05). (a–b) means that the same cheese sample stored for different months without common letters are significantly different (*P* < 0.05).

Dairy ingredients used for making PCs such as natural cheese and rennet casein were reported to contain proteases such as plasmin^32^, which might tolerate the thermal processing of PCs (at about 75 °C)^33^. The presence of active plasmin in PCs played a significant role in the protein hydrolysis of PCs during storage that affected the quality of cheese^31^. In this study, the plasmin activity was detected in all the PC samples during the storage (Fig. 2A), and higher plasmin activity of PC1 than that of PC2 and PC3 was observed. The higher plasmin activity of PC1 could be explained by the higher plasmin activity present in Cheddar cheese used. It was reported that Cheddar cheese contained higher plasmin activity than Mozzarella cheese since the hot-stretching process during processing of Mozzarella cheese inactivated the plasmin activity^9^. Moreover, all the PC samples showed a slight increase in plasmin activity during 4 months of storage (Fig. 2A). Correspondingly, decreased plasminogen activity in the PC samples during storage was observed (Fig. 2B). The increased plasmin activity in PCs during storage might be due to the conversion of plasminogen to plasmin by the plasminogen activator^34,35^.

Figure 2 shows the changes in the pH 4.6-soluble nitrogen (SN) (%) (Fig. 2C) and the intact casein contents (%) (Fig. 2D) in the PC samples during 4 months of refrigerated storage. Increased levels of pH 4.6-SN in all the PC samples were observed during the storage, and PC1 (from 2.97 ± 0.19% to 8.95 ± 0.41%) showed a higher level of pH 4.6-SN than PC2 (from 3.03 ± 0.15% to 7.20 ± 0.20%) and PC3 (from 2.59 ± 0.22% to 7.61 ± 0.40%). Correspondingly, the contents of intact casein in all the PC samples decreased significantly (*P* < 0.05) during the storage, and a lower content of intact casein in PC1 than those in PC2 and PC3 was observed (Fig. 2D). The higher level of pH 4.6-SN in PC1 and lower content of intact casein might be due to the higher proteolytic activities such as plasmin activity in PC1 than those in PC2 and PC3 as described above, though there was no significant difference between them (*P* > 0.05) (Fig. 2A). This suggested that other proteases, e.g. cathepsin B^36^, cathepsin D^37^, and total proteases^33^ in addition to plasmin in cheese might be involved in the protein degradation. Increased level of pH 4.6-SN with decreased content of intact casein due to continuous proteolysis during storage of processed cheese was also reported earlier^33^. PCs made with old aged Cheddar containing higher protease activity had lower content of intact casein than that made with young aged Cheddar^22^. As the degree of maturity of Cheddar cheese increased, the amount of intact casein in the cheese decreased by protein hydrolysis, thus there was reduced amount of intact casein in the PC made with the more aged Cheddar^38^. Furthermore, cheese analogues made with late-lactation rennet casein containing higher levels of plasmin were shown with higher levels of proteolysis than those made with mid-lactation rennet casein containing less plasmin^31^. Addition of more plasmin in milk used for making cheese also decreased the content of intact casein in the model cheese^39^.

### Fat particle size distribution in PC

**Figure 3:**
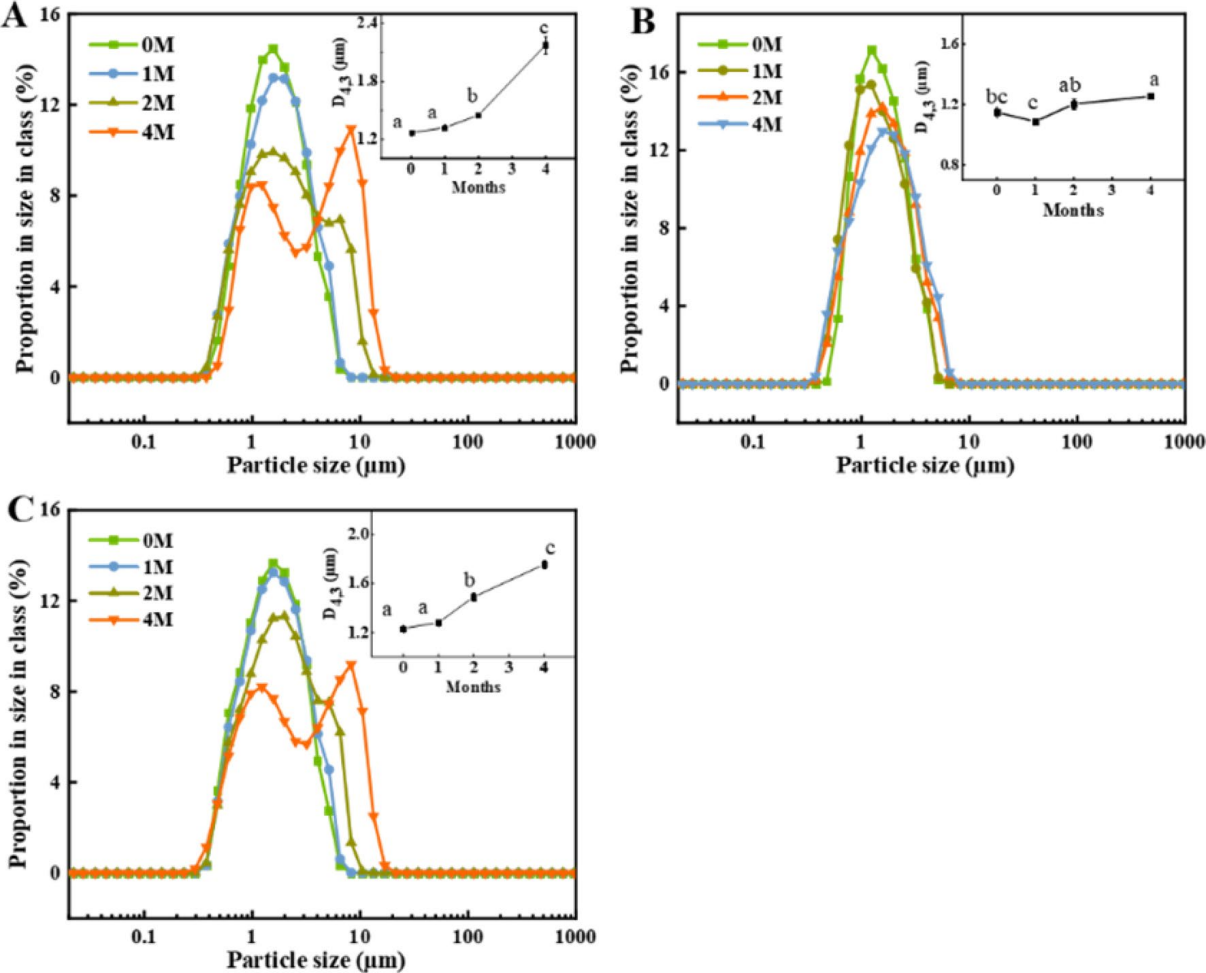
Change of fat particle size distribution in the processed cheeses made with Cheddar (PC1) (**A**), Mozzarella (PC2) (**B**) and both of them (1:1) (PC3) (**C**) during 4 months of refrigerated storage. The insert in panels shows the change in D[4,3] with different PCs. ^a-c^ means for the same cheese sample stored for different months without common letters are significantly different (*P* < 0.05).

The physical properties of PCs were directly influenced by the distribution, size and number of fat globules^40^. Figure 3A–C depicts the change of fat particle size distribution of the PC samples during 4 months of refrigerated storage. During the initial stage of storage (up to 1 month), the fat particle sizes of all the PC samples showed a narrow monomodal distribution, indicating that their fat droplets were distributed uniformly without coalescing. After 2 months of storage, the distribution ranges of PC1 (Fig. 3A) and PC3 (Fig. 3C) extended from 0.38 to 13.34 µm and 0.37 to 10.49 µm, respectively, suggesting that a small population of fat droplets in these PC samples had a tendency to congregate. In contrast, there were more uniform sizes for PC2 with narrow monomodal distributions during the whole period of storage. For all the PC samples, there was a significant (P < 0.05) increase in the average fat particle sizes determined as D^3,4^ of PC1 (2.18 µm), PC2 (1.25 µm) and PC3 (1.76 µm) after 4 months of storage when compared with those at the beginning of storage.

The difference in the fat particle size and distribution as observed in the 3 groups of PC samples at the beginning of storage could be mainly due to their different formulations and process parameters^20^. Further difference in the fat particle size distribution during the 4 months of storage might be resulted from the difference in the extents of protein hydrolysis in the PC samples^41^. The increase in the fat particle size with storage time might be due to the casein in the cheese absorbing water and swelling, which squeezed the fat globules to gradually consolidate^24^. Gonçalves et al.^12^ also found an increase in the fat globule size in natural Mozzarella cheese during the refrigerated storage.

### Moisture migration in PC

Low-field NMR was proven to be a rapid and non-destructive method for analyzing the moisture status of cheese^42^. The T_2_ relaxation time was utilized to assess the migration and state properties of water molecules in a cheese system^43^. Three T2 components were used: the peak within 0.1–1 ms (T_2b_) indicating the bound water by casein; the peak T_21_ from 10 to 100 ms indicating the entrapped water inside the casein network; the peak T_22_ from 100 to 1000 ms reflecting free water^44^,.

**Figure 4:**
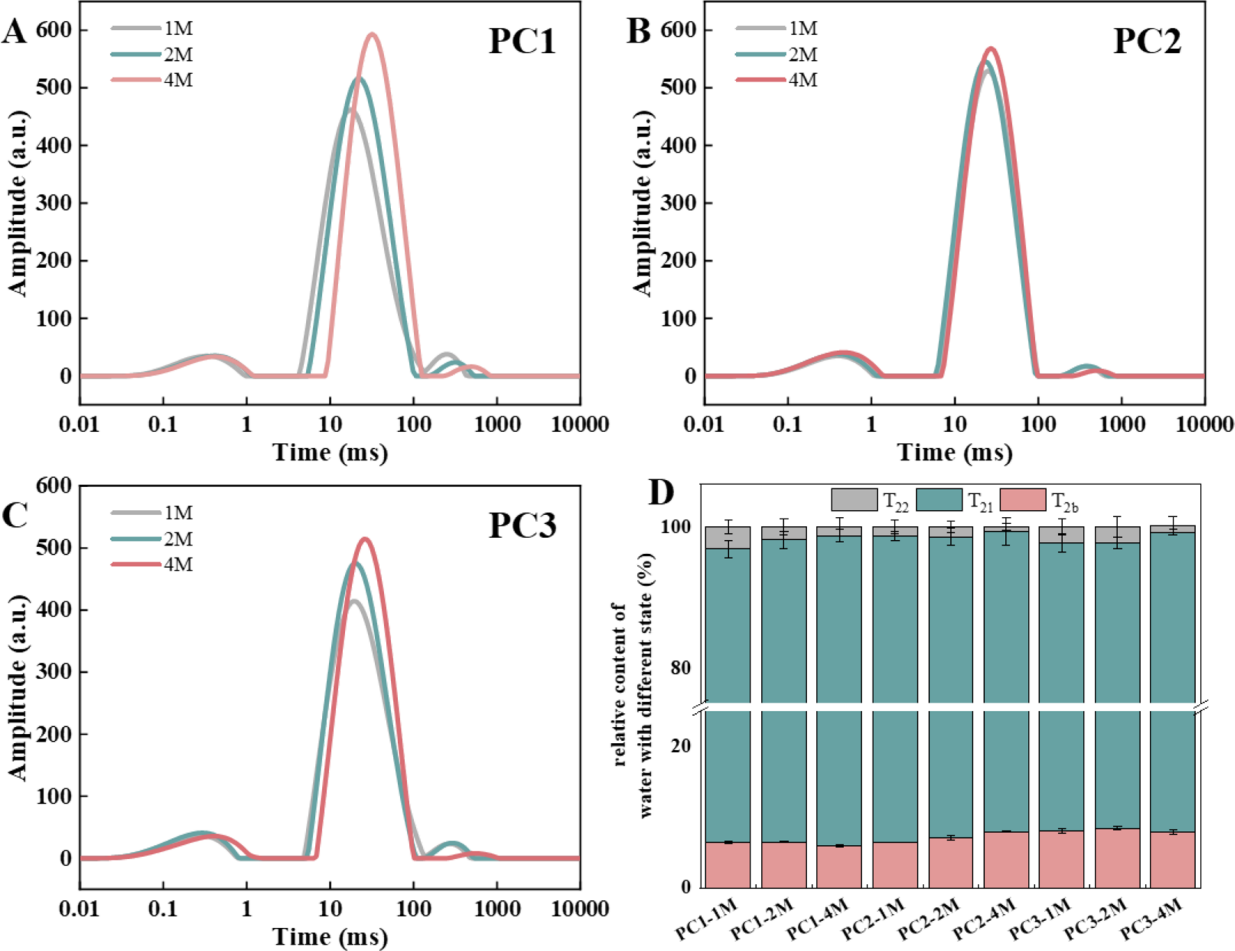
Changes of the T2 relaxation time distribution of the processed cheeses made with Cheddar (PC1) (**A**), Mozzarella (PC2) (**B**) and both of them (1:1) (PC3) (**C**), and relative content of different state of water in the PC samples (**D**) during 4 months of refrigerated storage.

The mobility and state of water molecules in the PC samples during the storage are shown in Fig. 4A–D. For all the samples, dominant entrapped water was present in the cheese matrix with rather small fractions of bound water and free water as indicated by the major T_21_ peak and very small T_2b_ and T_22_ peaks. Quantification of the different state of water (Fig. 4D) showed that the entrapped water accounted for more than 89% of the total water, and the free water content of PC1, PC2 and PC3 decreased by 59%, 48% and 58%, respectively, while there was little change of bound water for all the samples during 4 months of refrigerated storage. Correspondingly, a more obvious increase of the entrapped water in PC1 and PC3 than that in PC2 was also observed as indicated by the more increased amplitudes of the T_21_ peaks for PC1 (Fig. 4A) and PC3 (Fig. 4C) than that for PC2 (Fig. 4B) with the storage time. The increased entrapped water in PC1 and PC3 might be due to more protein hydrolysis in these samples (Fig. 2A). It was reported that cleavage of peptide bonds in casein produced more α-carboxylic acids and α-amino acids, which increased the water-binding capacity of casein and limited the proton mobility^24,32^. During the storage of PC, changes in molecular characteristics of casein with increased net negative charge and hydrophobic interactions between the molecules also promoted the formation of more compact casein aggregates that absorbed more water^9^.

### Confocal microscopy of PC

**Figure 5:**
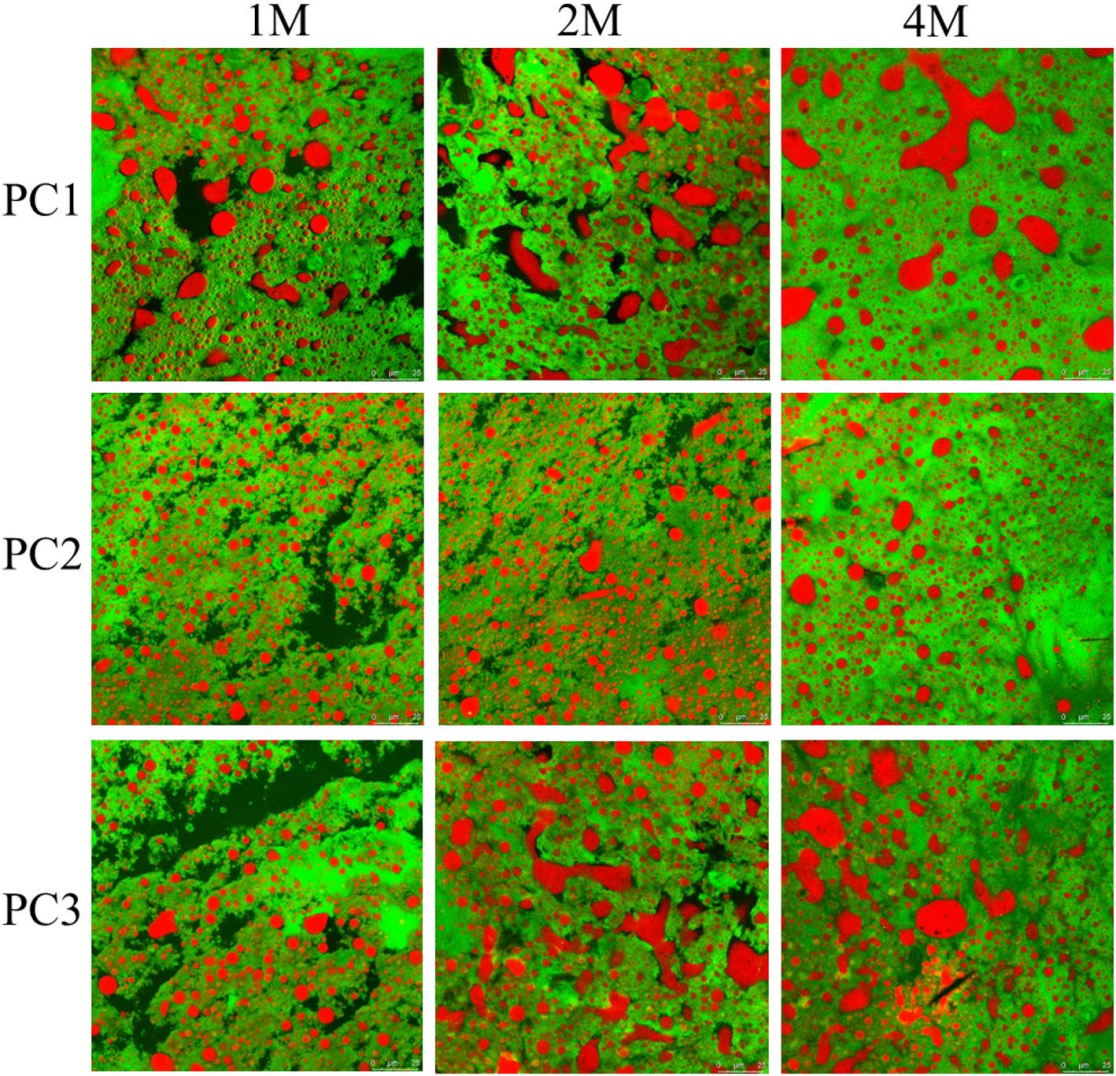
Confocal micrographs of microstructure of the processed cheeses made with Cheddar (PC1), Mozzarella (PC2) and both of them (1:1) (PC3) during refrigerated storage. 62 × magnification-scale bar is 20 µm (green = protein, red = fat & black = free water).

To look for insight into the microstructural changes of the PC samples during storage, confocal microscopy was used to assess the distribution of fat (labelled in red), protein (labelled in green) and aqueous phase (labelled in black) in PC (Fig. 5). In the first month, the 3 PC samples were shown with relatively small sizes of fat spheres in the protein matrix, and PC2 had a smaller size of fat with a more uniform distribution than PC1 and PC3. With prolonged storage for 2–4 months, increased sizes of fat and less (2 months) or non (4 months) aqueous phase were observed. This confirmed the observation described above (Fig. 4) that longer storage time might promote absorption of the free serum in the cheese matrix by the protein, causing more consolidation of fat globules with increased sizes. Previously, storage of the processed cheese analogue up to 60 days was observed with a gradual decrease of an initially large amount of free serum till it almost disappeared in the cheese matrix^33^. Hydrolysis of proteins into peptides during storage of PC was shown to facilitate binding of free serum present around protein and fat channels to the cheese matrix^45^. Furthermore, observation by scanning electron microscope of Mozzarella cheese over 40 days showed that the surface of the protein channels within the cheese changed from smooth to rough, suggesting migration of water into the protein matrix^24^.

## Conclusion

Changes in the physicochemical and functional properties of 3 groups of PC samples: PC1 made with Cheddar, PC2 with Mozzarella and PC3 with both at a ratio of 1:1, were compared during refrigerated storage for 4 months. All the PC samples had similar protein and moisture contents, but PC2 had lower fat content due to the lower fat content of the Mozzarella cheese used. PC2 showed decreased meltability and oil release than PC1 and PC3 which contained more fat. PC2 and PC3 exhibited better stretchability than PC1 which showed more protein degradation during the storage, though no significant (*P* > 0.05) difference in stretchability was observed between all the samples at the end of the storage. The difference in these changes of the PC samples during storage was confirmed by further analysis of the proteolytic activity, intact casein content, fat particle size distribution, moisture migration and microstructure of the samples. The present study suggested that the proteolytic properties and composition of natural cheese had a significant effect on the refrigerated storage stability of PC quality. Future studies will be carried out on the mechanism of the quality change of PC during storage as affected by the proteolytic activity originating from dairy and other ingredients used.

## Data Availability

All the data generated during this research study has been included in the manuscript.
